# Production and characterization of a new glycolipid, mannosylerythritol lipid, from waste cooking oil biotransformation by *Pseudozyma aphidis* ZJUDM34

**DOI:** 10.1002/fsn3.880

**Published:** 2019-02-17

**Authors:** Yongwu Niu, Jianan Wu, Wei Wang, Qihe Chen

**Affiliations:** ^1^ Department of Food Science and Nutrition Zhejiang University Hangzhou China; ^2^ Institute of Quality and Standard for Agriculture Products Zhejiang Academy of Agriculture Sciences Hangzhou China

**Keywords:** gas chromatography–mass spectrometry, mannosylerythritol lipids, response surface methodology, surface activity, waste cooking oil

## Abstract

Mannosylerythritol lipids (MELs) are glycolipids possessing unique biosurfactant properties. However, the prices of substrates currently used for MEL formation caused its unsustainable commercial development. Waste cooking oil poses significant ecological and economical problems. Thus, the production of MELs from used waste cooking oil using the biotransformation route is one of the better alternatives to utilize it efficiently and economically. This work aims at the production of MELs using waste cooking oil instead of soybean oil and evaluating the major characteristics and compositions of MELs. The titers reached 61.50 g/L by the optimization of culture medium, higher than the counterpart (10.25 ± 0.32 g/L) of the nonoptimized medium. MELs exhibited good surface activity and better performance in contrast to MELs grown on soybean oil. The water phase behavior of MEL‐A was also evaluated. The process showed higher productivity of MELs with better surface activity and application stability than the conventional process using soybean oil. The findings of this study imply that the use of inexpensive fermentation substrates associated with straightforward downstream processing is expected to have a great impact on the economy of MEL production.

## 1 INTRODUCTION

**Figure 1:**
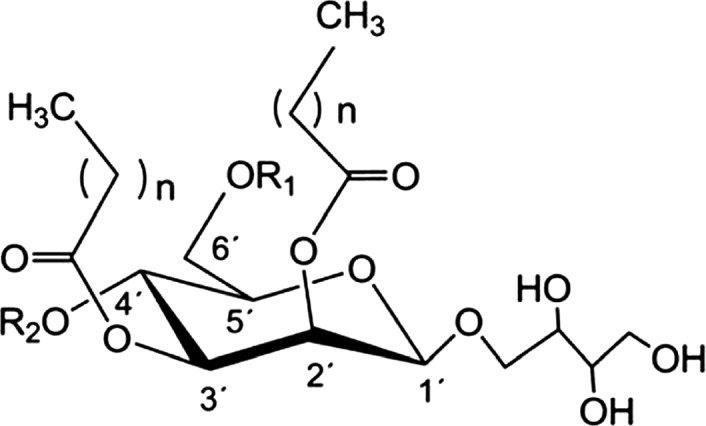
General structure of mannosylerythritol lipids (MELs). MEL‐A: R1/R2=acetyl‐; MEL‐B: R1=acetyl‐, R2=H; MEL‐C: R1=H, R2=acetyl‐; MEL‐D: R1/R2=H

Mannosylerythritol lipids (MELs) are one of the most promising nonionic biosurfactants, secreted by plant‐associated fungi of the genera *Pseudozyma* and *Ustilago*(Arutchelvi, Bhaduri, Uppara, & Doble, 2008; Yu et al., 2015). MELs are amphiphilic molecules with 4‐*O*‐β‐d‐mannopyranose‐erythritol as a hydrophilic moiety and a fatty acid and/or an acetyl group as the hydrophobic moiety (Arutchelvi & Doble, 2011). According to the different number and location of acetyl group at mannosyl C‐4 and C‐6, MELs are classified as MEL‐A, MEL‐B, MEL‐C, and MEL‐D (Figure 1; Gunther et al., 2015). MELs were first noted as oily compounds in the culture suspension of *Ustilago maydis*(Haskins, Thorn, & Booroyd, 1955). Though MELs and their properties have been documented for several decades, they gained more and more attention only in recent years because of their interesting application possibilities in the biochemical and pharmaceutical industries. Up to date, researches have shown that MELs are mainly produced by anamorphic basidiomycetous yeasts, *Pseudozyma*spp., as relatively high quantities, and fungi, *Ustilago maydis*, as relatively low quantities (Faria et al., 2014), which is with regard to the substrate, fermentation condition, and downstream processing.

In contrast to other biosurfactants, for example, rhamnolipids and sophorolipids, MELs own many excellent physicochemical properties such as good emulsifying properties, biodegradability, and lower critical micelle concentration (CMC), but also many special physiologic activities, such as inhibiting the growth of microorganisms (Kitamoto et al., 1993), inducing cell mutation (Isoda & Nakahara, 1997; Wakamatsu et al., 2001), differentiate human myeloid leukemia cell lines and melanoma cells (Fan, Li, Niu, & Chen, 2016; Zhao et al., 2000, 2001), improving the efficiency of gene transfection (Kitamoto, 2008), and a strong coordination ability with glycoproteins (Fan, Xie, Wang, Huang, & Zhou, 2018; Im, Nakane, Yanagishita, Ikegami, & Kitamoto, 2001; Im et al., 2003); thus, MELs could be used in lots of industries (Morita, Fukuoka, Imura, & Kitamoto, 2013; Noh, Suh, & Park, 2016; Safdel, Anbaz, Daryasafar, & Jamialahmadi, 2017), for example, environmental protection, food, cosmetic, and pharmaceutical.

Until now, noncommercial MEL products rarely appeared. One of the main reasons is high production costs, which is related to the expensive raw materials, low production, and tough downstream, etc. Among them, several substrates have been used for MEL production by *Pseudozyma*spp*.*, including soybean oil, alkanes, glycerol, glucose, and xylose. (Arutchelvi & Doble, 2011; Morita, Fukuoka, Imura, & Kitamoto, 2013; Rau, Nguyen, Schulz et al., 2005). In addition, almost all vegetable oils (except palm oil and coconut oil) have been found to serve as a good carbon source for MELs by different *Pseudozyma*spp*.* However, the sustainability of MEL production from the above‐mentioned materials is doubtful due to high cost (Faria et al., 2014). Consequently, a positive strategy to reduce the production cost is to search for cheap substrates. So far, biosurfactant that has been shown could be produced using some renewable industrial residues such as olive oil mill effluent (Mercadé et al., 1993), oil refinery wastes (Bednarski, Adamczak, Tomasik, & Plaszczyk, 2004), distillery and whey wastes, potato process effluent (Dubey & Juwarkar, 2001), soapstock (Benincasa, Contiero, Manresa, & Moraes, 2002), and waste cooking oil (Raza, Khan, Khalid, & Rehman, 2006). Waste cooking oil is generated in large quantities during food preparation in both household and food processing industries (Hingu, Gogate, & Rathod, 2010; Zhang, Wang, & Mortimer, 2012). It has been considered a problematic waste product contributing to the environmental pollution. Currently, waste cooking oils are only allowed to produce biofuels. In general, the disposal of waste cooking oil is a growing problem that needs effective solution.

Biosurfactant production using the technique of microorganism transformation may be an effective way to utilize waste cooking oil. There were some studies on glycolipid production using waste cooking oil. Waste frying oil and the methyl ester of coco/palm oil were used to produce sophorolipids by *Candida bombicola* in shaking flasks (Fleurackers, 2006). Haba, Espuny, Busquets, and Manresa (2000) reported the reuse of olive and sunflower cooking oil as substrate to produce rhamnolipids at equivalent productivity of 6.75–9.25 g/L (measured as 2.7 g rhamnose/L) in shaking flasks. Waste frying oil as the sole carbon source was used to synthesize rhamnolipids by *Pseudomonas aeruginosa* ZJU at a concentration of 12.47 g/L, and its mutant after treatment by UV light increased this productivity to 24.61 g/L cultured in shaking flasks. Future, the productivity of fermentation which conducted in a 50‐L bioreactor reached over 20 g/L (Zhu et al., 2007).

There is no report on the MEL production from waste cooking oil, while Włodzimierz Bednarski, Adamczak, and Nawotka (2006) studied on the synthesis of MELs from glucose and products of enzymatically hydrolyzed lactose in milk permeate (after ultrafiltration) supplemented with the waste fats (fish, pork, and postrefining fatty acids), indicated that the fatty acids from the waste fats used seemed to be directly incorporated in the mannosylerythritol lipids.

To obtain higher MEL yield, the fermentation conditions have been widely studied, showing that the type and concentrations of nitrogen and inorganic salts, the amount of vegetable oil, inoculum size, and initial pH are potentially important impact factors (Luo et al., 2013). In this work, waste cooking oil was used as the sole carbon source to produce MELs by *Pseudozyma aphidis*ZJUDM34. The aim of this work was to use response surface methodology (RSM) to optimize the amount of waste cooking oil, inoculum size, initial pH, and medium volume for MEL production. Furthermore, to demonstrate the surface activity and stability, MELs from waste cooking oil were compared to that from soybean oil via the analyses of gas chromatography–mass spectrometry (GC‐MS) and surface tension examinations.

## 2 METHODS

### 2.1 Fermentation strain

The experimental strain was *P. aphidis*DSM 70725, which obtained from Deutsche Sammlungvon von Mikroorganismen und Zellkulturen GmbH (DSMZ), Braunschweig, Germany. The *P. aphidis* ZJUDM34 mutant was evolved from wild‐type yeast using UV mutagenesis and low‐energy N^+^ implantation methods. It exhibited enhanced ability to produce MEL‐A. This mutant was stored in our laboratory. Stock cultures were grown for 2 days at 28°C in a liquid medium containing (g/L) yeast extract 3.0, malt extract 3.0, peptone from soybeans 5.0, and glucose 10.0 at initial pH and then mixed with an equal volume of 50% glycerol. They were stored at −80°C and renewed every half‐year.

**Figure 2:**
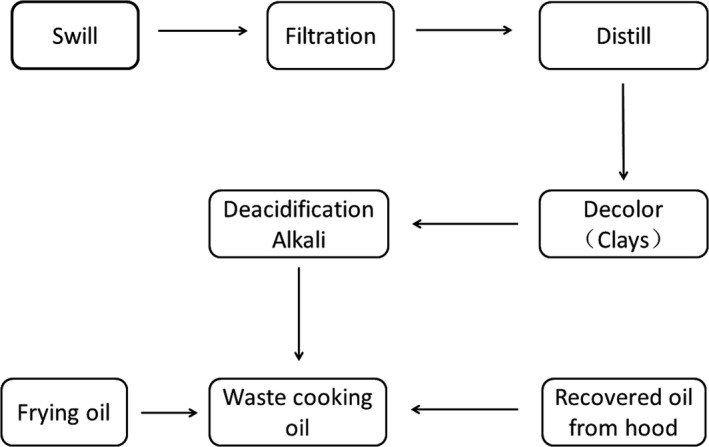
The preparation route of waste cooking oil

Waste cooking oil was collected from major restaurant in Hangzhou with soybean oil in the preparation of cooked food. The crude waste oil was preliminarily prepared using the process as shown in Figure 2, prior to use as the carbon source in replace of soybean oil.

### 2.2 Inoculum preparation

At first, the yeast was activated by inoculating 1‐ml stock cultures into 50‐ml YM medium composed of (g/L): yeast extract 3.0, malt extract 3.0, peptone from soybeans 5.0, and glucose 10.0 at the natural pH in 250‐ml Erlenmeyer flasks, and was then incubated for 36 hr at 28°C and 180 rpm. Seed culture was prepared by inoculating 1 ml of the activated cells in 250‐ml Erlenmeyer flasks containing 50‐ml culture medium (g/L) (NaNO_3_ 3, KH_2_PO_4_ 0.3, MgSO_4_·7H_2_O 0.3, yeast extract 1, and glucose 40) and incubated for 2 days at 28°C and 180 rpm. Then, the inoculum cells were prepared by the following procedure: Seed was centrifuged to obtain the cells, which were washed two times with 0.9% physiologic saline, dissolved in an appropriate amount of 0.9% physiologic saline to formulate into the inoculum with the amount of 0.12 g/ml cell wet weight.

### 2.3 Fermentation medium and conditions

The liquid submerged fermentation (g/L) medium was comprised of NaNO_3_ 3.0, KH_2_PO_4_ 0.3, MgSO_4_·7H_2_O 0.3, yeast extract 1.0, and certain amount of waste cooking oil (ml/L) or soybean oil (ml/L) depending on experimental design. Fermentation was inoculated with the above inoculum and conducted in 250‐ml Erlenmeyer flasks for 10 days at 28°C and 180 rpm.

### 2.4 Experimental design

Response surface methodology is a statistical method to find the optimal process parameters to solve multivariable problems with multiple regression analysis for the quantitative data obtained from properly designed experiments. In order to obtain optimum conditions for increasing MELs’ yield, a central composite design with five coded levels was performed. In the experimental design, four factors (waste cooking oil amount, inoculum size, medium volume in 250‐ml Erlenmeyer flasks, and initial pH) that having impact on the production of MELs were identified by the optimization strategies.

All the experiments were performed in triplicate. The test factors were coded according to the following equation (Zhang, Dong, Fan, Jiao, & Chen, 2015):

(1) $$x_{i}=\frac{X_{i}-X_{0}}{\Delta X_{i}}$$

where *x_i_* is the coded value of the *i*th independent variable, *X_i_* is the natural value of the *i*th independent variable, *X*_0_ is the natural value of the *i*th independent variable at the center point, and Δ*X_i_* is the step change value. The range and the levels of the test factors with both coded values and natural values investigated in this study are given in Table 1. The production of MELs was considered as the response.

**Table 1:** Range of values for response surface methodology

Independent variables	Variable name	Levels^a^				
		−2	−1	0	1	2
*X*_1_ (ml/L)	Waste cooking oil amount	54.8	65.0	80.0	95.0	105.2
*X*_2_ (ml/L)	Inoculum size	11.6	15.0	20.0	25.0	28.4
*X*_3_ (ml/250 ml)	Medium volume	24.77	35.00	50.00	65.00	75.23
*X*_4_	Initial pH	3.41	4.43	5.93	7.43	8.45

a *x*_1_ = (*X*_1_ − 80)/15; *x*_2_ = (*X*_2_ − 20)/5; *x*_3_ = (*X*_3_ − 50)/15; *x*_4_ = (*X*_4_ – 5.93)/1.5.

The quadratic model for predicting the optimal point was expressed based on the following equation:

(2) $$Y=b_{0}+\sum b_{i}x_{i}+\sum b_{\mathrm{ii}}x_{i}^{2}+\sum b_{\mathrm{ij}}x_{i}x_{j}$$

where *Y* was the response variable, *b*_0_, *b_i_*, *b_ii_*, and *b_ij_* were the regression coefficients variables, for intercept, linear, quadratic, and interaction coefficients, respectively, and *x_i_* and *x_j_* were independent variables. Results were analyzed using the response surface regression (RSREG) procedure (SAS Institute Inc, Cary, NC, USA).

Three‐dimensional surface plots were drawn to illustrate the main and interactive effects of the independent variables on MEL production. Solving the regression equation and analyzing the response surface contour plots, the optimum values of the selected variables were obtained for the further experiments (Ghorbani, Karimi, Biria, Kariminia, & Jeihanipour, 2015).

To make clear the fermentation process, according to the optimal results of 2.3.1 experiments, design experiments to conduct the assay of biomass, pH, and MEL yield at different culture times. Selected measurement time points were the 3rd, 4th, 5th, 6th, 7th, 8th, 9th, and 10th day.

### 2.5 Determination of biomass, isolation, and purification of MELs

After fermentation, pH was measured with pH meter. Then, thoroughly mixed culture suspension with equal volume of ethyl acetate was extracted three times. Aqueous and organic phases were separated by centrifugation, with the yeast cells between the two phases. The collected organic phases were rotary evaporated (45–60°C, 3,000 Pa) to obtain crude MELs, and the yeast cells were collected in weighed dry flats which were put in the oven to constant weight for measurement of biomass (g dry cells/L fermentation broth). Then, the crude MELs were treated three times by solving the extract in methanol–cyclohexane (v:v, 1:1). The methanol phases were collected and again rotary evaporated, obtained the purified MEL product containing minor amounts of oil (Rau, Nguyen, Roeper, Koch, & Lang, 2005).

### 2.6 Thin‐layer chromatography

The purified MEL extracts were analyzed by thin‐layer chromatography (TLC). As a solvent system, a chloroform–methanol–water (65:15:2) mixture was used. Location of the components on the plates was carried out by fumigating with iodine or spraying the plates with a solution of 0.1% orcinol in a 5% sulfuric acid solution and heating the plates for 5 min at 110°C.

### 2.7 Gas chromatography–mass spectrometry

#### 2.7.1 Methyl ester of samples

In this study, the methyl ester derivatives were prepared by the followed steps. The purified MEL fraction or oil sample (2.0 g) was mixed with 20 ml methanol and 0.5 ml KOH (1 M in methanol) in round bottom flask, then added a few zeolite, and begun to heat reflux with shaking the beaker, until the solution was clear. Afterward, the solution was cooled in cold water, transferred to a separating funnel, washed the flask with 10 ml *n*‐heptane and transferred to the separating funnel, added to 20 ml distilled water, shook and still layered; the upper is ester layer, and the lower is aqueous layer. The lower layer was treated with 10 ml *n*‐heptane extraction and mixed with the upper. Subsequently, the *n*‐heptane solution was washed several times with distilled water until the water was neutral; acetate layer was obtained, dried with anhydrous sodium sulfate, filtered, and evaporated to 10 ml *n*‐heptane solution for analysis.

#### 2.7.2 Determination procedures of GC‐MS

About 3 ml of the upper phase from the isolated MELs or soybean oil was injected into the GC‐MS system (Agilent 6890 Gas Chromatography with a 5973 Mass Spectrometer) using an Agilent HP‐5MS (30 m × 0.25 mm×0.25 μm) column. The oven temperature was programmed from 100°C to 300°C within 5 min. A scan range of 30–550 u was applied, using a source temperature of 250°C (Onghena et al., 2011).

### 2.8 Determination of surface activity and stability of MELs

#### 2.8.1 Surface‐activity determination

Equilibrium surface (water/air) tension measurements of MEL solution were performed at 25°C with a QBZY‐1 tensiometer (Fangrui Instrument Co., Shanghai, China) using the Wilhelmy plate method (Benincasa et al., 2002). To increase the accuracy of the surface tension measurements, an average of triplicates was determined. The platinum plate and all glassware used were cleaned in ultrapure water.

#### 2.8.2 The critical micelle concentration and stability

Critical micelle concentration is the concentration of an amphiphilic component in solution at which the formation of micelles is initiated. It is important for some biosurfactant applications. The CMC was determined by plotting the surface tension as *y*‐axis versus the concentration of MELs as *x*‐axis and was found at the break point. Aqueous solutions of MELs (in the concentration range of 1.50–100 mg/L) were obtained by successive dilutions of a concentrated sample prepared by weight in ultrapure water (Gudina, Teixeira, & Rodrigues, 2010).

The applicability of biosurfactants can be conditioned by their stability to pH changes, temperature range, and salt tolerance. Thus, we evaluated the stability of MELs under different environmental conditions. The MEL solutions with CMC were prepared for evaluating the stability. Then, the stability of the MELs was determined by measuring the surface tension with different pH values (2.00–12.00). Furthermore, the MEL solutions were treated at different temperatures (−20°C, 0°C, 50°C, 65°C, 80°C, and 95°C) for 1 or 2 hr and evaluated the stability by assaying the surface tension. Lastly, the surface tension of MEL solutions with different amounts of sodium chloride was assayed for evaluating salt tolerance. Surface tension of each sample was determined as described above, and all measurements were performed in triplicate.

### 2.9 Statistical analysis

The regression analysis of the obtained experimental data was performed by Design‐Expert software (Stat‐Ease Inc., Minneapolis, MN, USA). The quality degree of the fit for the polynomial model equation is expressed as the coefficient of determination R2, and its statistical significance was checked by the *F* test. Three independent experiments were carried out for standard error analysis of the mean (*SEM*), which was analyzed by Origin 9.1 software (OriginLab Corp., Northampton, MA, USA). Data were expressed as means ± *SEM*. The error bars in the figures represent the standard error of the mean.

## 3 RESULTS

### 3.1 Optimization under RSM‐based experimental design

Previous investigations showed that waste cooking oil, inoculum size, culture medium volume, and initial pH all affected the production of MELs by *P. aphidis*ZJUDM34. Thus, to further improve the fermentation conditions for MEL production, RSM was employed to determine the optimal levels of four factors (waste cooking oil amount, inoculum size, medium volume in 250‐ml Erlenmeyer flasks, and initial pH) that affected MEL production (Table 2). As a result, the following regression equation was obtained:

(3) $$Y=17.24x_{1}+8.32x_{2}-0.39x_{3}+1.19x_{4}+4.30x_{1}^{2}+2.53x_{2}^{2}-2.88x_{3}^{2}-3.82x_{4}^{2}+12.56x_{1}x_{2}-1.15x_{1}x_{3}-2.33x_{1}x_{4}+2.80x_{2}x_{3}+3.40x_{2}x_{4}-2.09x_{3}x_{4}+17.99$$

**Table 2:** Experimental design and the results of response surface methodology

Run	*x*_1_	*x*_2_	*x*_3_	*x*_4_	MELs (g/L) observed	MELs (g/L) predicted
1	1	1	1	−1	55.38	57.35
2	0	0	1.68	0	13.29	50.65
3	0	0	0	0	14.00	3.50
4	0	0	0	0	10.00	2.08
5	0	0	0	0	8.00	16.36
6	1	1	−1	−1	51.43	5.04
7	0	0	0	0	10.00	5.04
8	1	−1	1	1	1.54	4.94
9	0	−1.68	0	0	12.00	1.16
10	0	0	0	1.68	10.00	59.16
11	−1	−1	1	−1	3.08	11.16
12	−1.68	0	0	0	2.00	39.16
13	0	0	0	0	10.00	10.50
14	0	1.68	0	0	40.00	9.19
15	−1	1	1	1	3.08	5.16
16	0	0	−1.68	0	8.07	9.16
17	0	0	0	−1.68	6.00	17.99
18	1	−1	−1	1	17.14	17.99
19	1.68	0	0	0	60.00	57.99
20	−1	1	−1	1	2.86	17.99
21	−1	−1	−1	−1	5.71	11.99

Note

MEL: mannosylerythritol lipid.

With *Y*, MEL production (the response); *x*_1_, waste cooking oil amount; *x*_2_, inoculum size; *x*_3_, medium volume in 250‐ml Erlenmeyer flasks; and *x*_4_, initial pH (coded values). The significance of each coefficient was determined by *p*‐values, which are listed in Table 3.

**Table 3:** Model coefficients estimated by multiple linear regression

Factor	Coefficient estimate	*F* value	*p*‐Value
Model	17.99	19.06	0.0004^a^
*x*_1_	17.24	69.75	<0.0001^a^
*x*_2_	8.32	16.25	0.0040^a^
*x*_3_	−0.38	0.08	0.7549
*x*_4_	1.19	0.33	0.5416
$x_{1}^{2}$	4.30	11.47	0.0016^a^
$x_{2}^{2}$	2.53	3.98	0.0080
$x_{3}^{2}$	−2.88	5.14	0.4085
$x_{4}^{2}$	−3.83	9.09	0.1350
*x*_1_ *x*_2_	12.56	21.69	0.0020^a^
*x*_1_ *x*_3_	−1.15	0.44	0.4831
*x*_1_ *x*_4_	−2.33	0.75	0.3684
*x*_2_ *x*_3_	2.80	2.60	0.1200
*x*_2_ *x*_4_	3.40	1.59	0.2068
*x*_3_ *x*_4_	−2.09	1.45	0.2255
Lack of fit		0.92	0.4700

a Statistically significant at 95% of confidence level.

The regression equation indicated the *R* ^2^ value of 0.9780. This value ensured a satisfactory adjustment of the quadratic model to the experimental data and indicated that the model could explain 97.80% of the variability in the response. As can be seen from Table 3, the *p*‐value and lack of fit check indicated that the model was significant for MEL production. As well, the production was significantly influenced by the variables such as waste cooking oil amount and inoculum size, independently. Moreover, there were interactions among *x*_1_and *x*_2_ factors (*p* < 0.05). However, other factors demonstrated insignificant effects on the production of MELs.

**Figure 3:**
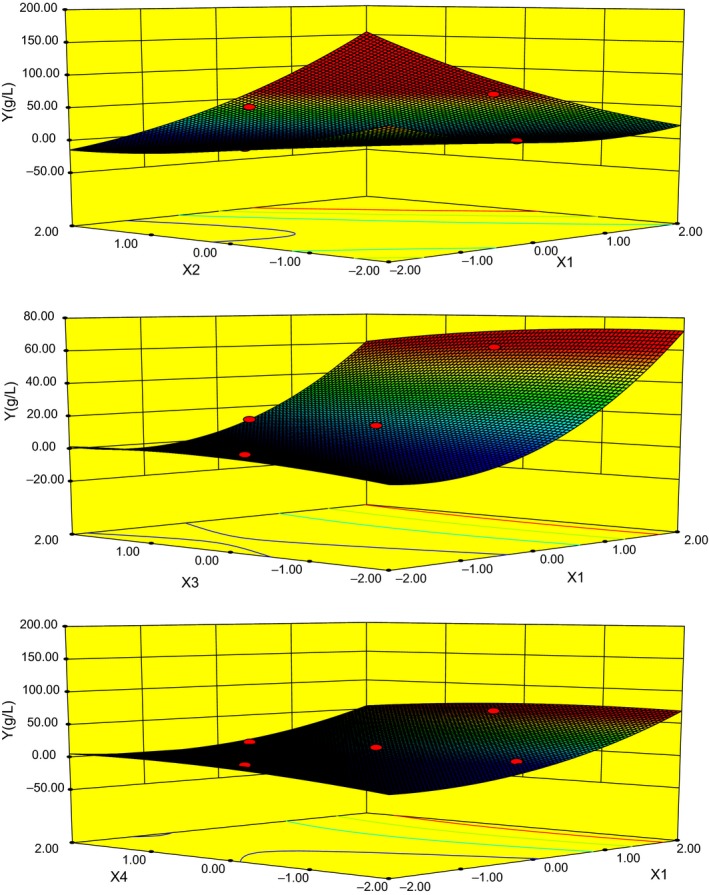
Response surface plots of *x*_1_, *x*_2_, *x*_3_, and *x*_4_ against MEL (g/L) production by *Pseudozyma aphidis* ZJUDM34

The three‐dimensional plot obtained from the calculated response surface is presented in Figure 3. Three‐dimensional response surface plots of four factors against MEL production (*Y*) can explain the results of statistical and mathematical analyses. The plot clearly showed that *Y* reached its maximum at a combination of coded level −1(*x*_1_), 0.99(*x*_2_), −0.09(*x*_3_), −0.05(*x*_4_). The predicted maximum production of MELs is 61.51 g/L, which is much higher than the yield from the control fermentation (10.25 ± 0.32 g/L).

### 3.2 Experimental validation of the optimized culture conditions

**Figure 4:**
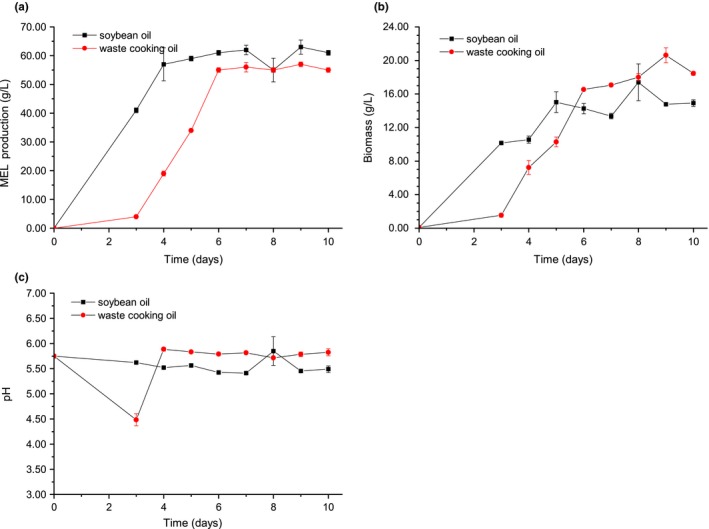
Mannosylerythritol lipid (MEL) production from soybean oil or waste cooking oil by *Pseudozyma aphidis* ZJUDM34. (a–c) Time course showing MEL formation yield, biomass, and pH from soybean oil (black square) or waste cooking oil (red circle) with Table 4 conditions by *P. aphidis*, respectively

The predicted optimal fermentation conditions for MEL production composed of waste cooking oil amount 95 ml/L, 24.95 inoculum size (ml/L), 48.65‐ml medium volume per 250 ml, and initial pH 5.76. The response surface plots are presented in Figure 3. In order to validate the adequacy of this optimized medium formula for MEL production, verification experiments were carried out at the predicted optimal soybean oil and waste cooking oil. The mean output of MELs from waste cooking oil was 55.00 ± 0.80, which was little lower than the predicted value (61.51 g/L) and slightly lower than 61.00 ± 0.80 g/L from soybean oil after 10 days (Figure 4a). In comparison, waste cooking oil showed lower MEL production and biomass than those of soybean oil (Figure 4b), whereas there was no obvious change of pH during the fermentation process (Figure 4c). These results suggested that the model obtained from RSM was adequate and waste cooking oil was a suitable carbon source for MEL production by *P. aphidis*ZJUDM34.

### 3.3 Fatty acid profiles in substrates and MELs

**Figure 5:**
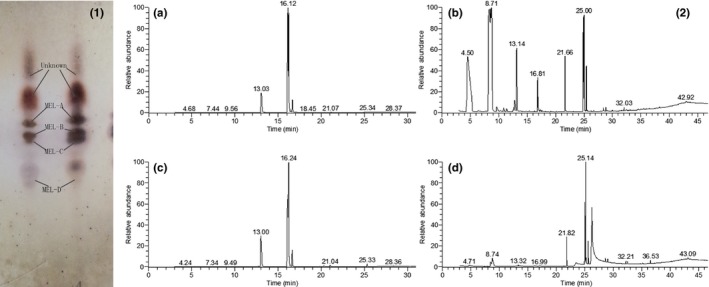
(1) Thin‐layer chromatography of mannosylerythritol lipid (MEL) obtained from liquid–liquid extraction with ethyl acetate of culture broth samples (after 10 days, left: MELs from soybean oil; right: MELs from waste cooking oil); (2) The total ion chromatogram of oil samples and MELs by means of gas chromatography–mass spectrometry. (a) Soybean oil sample; (b) waste cooking oil sample; (c) MELs derived from soybean oil; (d) MELs from waste cooking oil

Furthermore, by the use of TLC, MELs contain type‐A, type‐B, and type‐C grown on soybean oil and type‐A, type‐B, type‐C, and type‐D from waste cooking oil (Figure 5(1)). Soybean oil and waste cooking oil as the substrates were composed of a variety of fatty acids and likely influenced the properties of MELs; as a result, GC‐MS was used for detecting fatty acid contents of substrate and MEL product. To compare the fatty acid profiles of soybean oil, waste cooking oil and a mixture of MEL derived from soybean oil or waste cooking oil are presented in Table 4 and Figure 5(2). The substrate, soybean oil, included C16:0, C16:1, C17:0, C18:0, C18:0, C18:1, C18:2, C18:3, C20:0, and C22:0, and waste cooking oil had similar fatty acid composition to soybean oil except for lack of C18:3 and a little additional C14:0, C20:1, and C20:2. In contrast, there is more C18:2 (48.11%) in soybean oil than waste cooking oil (30.63%). However, these two oils showed no significant difference in the overall fatty acids. For MELs grown on soybean oil, main fatty acid compositions are C6:0 (23.19%), C10:0 (47.49%), C12:0 (5.94%), C18:1 (7.37%), and C18:2 (9.57%), and the MELs from waste cooking oil were composed of C10:0 (6.66%), C16:0 (7.92%), C18:1 (59.78), and C18:2 (13.78%). This result indicated soybean oil contributed to more short‐chain fatty acids in MEL production, while waste cooking oil showed favorable for producing more long‐chain fatty acid.

**Table 4:** Main fatty acid contents (percent of main fatty acid, MFA) in feedstock and fermentation products

Fatty acid chain	Fatty acid chain in each sample composition (%)			
	Soybean oil	Waste cooking oil	MELs (SO)	MELs (WCO)
C8:0	—	—	23.19	1.50
C9:0	—	—	0.10	—
C10:0	—	—	47.49	6.66
C10:1	—	—	0.70	3.22
C11:0	—	—	0.42	—
C12:0	—	—	5.94	0.31
C12:2	—	—	0.07	—
C14:0	—	0.04	0.05	0.02
C16:0	13.08	18.59	2.13	7.92
C16:1	0.06	0.37	—	—
C17:0	0.02	0.04	—	—
C18:0	4.36	5.13	1.60	3.79
C18:1	33.60	43.57	7.37	59.78
C18:2	48.11	30.63	9.57	13.78
C18:3	0.51	—	—	1.92
C20:0	0.13	0.60	0.13	0.55
C20:1	—	0.43	0.16	—
C20:2	—	0.05	—	—
C20:3	—	—	—	0.06
C20:4	—	—	0.95	—
C22:0	0.13	0.56	0.13	0.48

Note

—: not detected; MEL: mannosylerythritol lipid; WCO: waste cooking oil.

### 3.4 Surface activity and stability

#### 3.4.1 Critical micelle concentration

**Figure 6:**
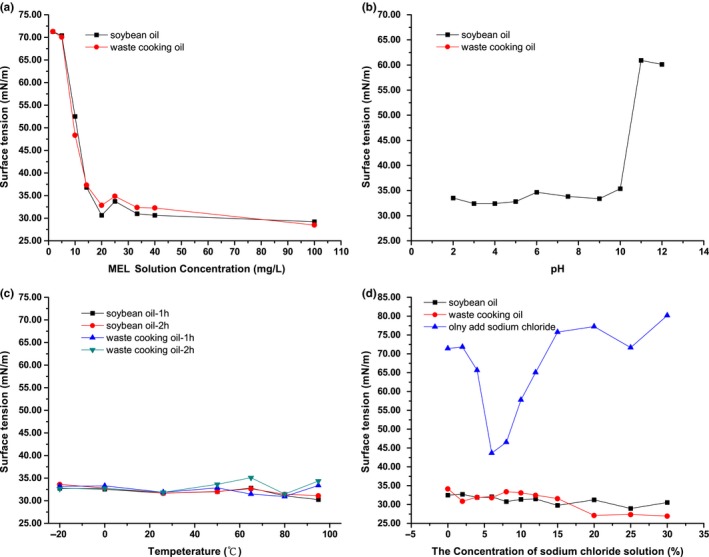
The critical micelle concentration and stability to pH, temperature, and sodium chloride of MELs from soybean oil or waste cooking oil by *Pseudozyma aphidis*. (a) Surface tension (mN/m) of the crude MELs obtained from soybean oil (black square) or waste cooking oil (red circle) by *P. aphidis*dissolved in ultrapure water at different concentrations at 25°C; (b) effect of pH (2.00–12.00) on the surface tension of crude MELs; (c) effect of temperature (−20.0°C to 95.0°C) with different treatment times (1 or 2 hr) on the surface tension of crude MELs; (d) effect of sodium chloride concentration (m/v, 0.0%–30.0%) on the surface tension of ultrapure water and crude MEL solutions. Samples were prepared with a concentration of 20.00 mg/ml, and measurements were done at 25°C. The reference surface tension value was 71.97 mN/m. Results represent the average of three independent measurements

The surface (water/air) tensions of crude MEL from different substrates were comparatively measured. As shown in Figure 6a, the strongest reduction (30.63 or 32.83 mN/m) of the surface tension caused by 20.00 mg/L MEL was separately achieved with the product from soybean oil or waste cooking oil. Besides, MEL‐A was of two CMCs; thus, the surface tensions became a little higher with the concentration of MELs from 20.00 to 33.33 mg/L (Imura et al., 2006). Thus, the CMCs of MELs grown on soybean oil or waste cooking oil were both 20.00 mg/L.

#### 3.4.2 The stability of MELs to pH, temperature, and sodium chloride solution

To evaluate the pH effect on stability of MELs from different substrates, the surface tension of several MEL samples prepared with a concentration of 20.00 mg/ml at different pH values (2.00–12.00) was determined. As can be seen from Figure 6b, the surface tension values were between 30.00 and 35.00 mN/m as pH less than 10.00. However, if pH is higher than 10.00, the surface tension rapidly rises up to about 60.00 mN/m, which indicated that MELs from soybean oil or waste cooking oil were unsuitable at high alkali environmental condition. As presented in Figure 6c, both MELs produced from soybean oil and waste cooking oil remain the unaltered activity after incubation for 2 hr at −20°C, 0°C, 50°C, 65°C, 80°C, 95°C, and room temperature. Therefore, it can be concluded that the surface properties of MELs are stable within −20°C to 95°C.

Further, the salt tolerance of MELs was explored, as the results presented in Figure 6d, MELs from soybean oil and waste cooking oil showed constant surface tensions and even a downward trend as the salt concentration increases, which indicated that both of MELs retained good surface activity at high salt environment.

## 4 DISCUSSIONS

The aim of this work was to investigate the use of waste cooking oil for MEL production by *P. aphidis* ZJUDM34. The technical feasibility of the transforming waste cooking oil into MELs was demonstrated. The novel fermentation strategy for MEL production was developed by optimization using RSM. The novel MELs were further characterized through the comparative analyses of its surface property to that produced from soybean oil. In term of the fermentation capability and the major properties, it is obvious that the replacement of soybean oil with waste cooking oil (WCO) is feasible for MEL production. The choice of cheap raw materials is vital to overall economics of the fermentation process since they account for nearly 50% of final production cost (Makkar & Cameotra, 1999). Moreover, in contrast to waste cooking oil, the average price of soybean oil was up to about three times (Yaakob, Mohammad, Alherbawi, Alam, & Sopian, 2013). The finding of this study showed the complete replacement of fermentation substrate with waste oil for MEL production is feasible and economic.

In consideration of the reuse of waste used oil, the industrial significance is rather high. Presently, it was reported that world production of oils and fats is above 200 million tonnes, 81% of which are derived from plants (Smith, 2004). Most of the oils and fats are used in the food industry and other consumed factories, which generates great quantities of waste oils. Naturally, the disposal of waste cooking oil is a growing problem, which explains the increasing interest in the use of waste oils for microbial transformation. Fleurackers (2006) reported the use of waste frying oils for the production of sophorolipids at 50 g/L medium by *Candida bombicola*, which was comparable to the yield of more commonly used carbon sources such as oleic acid. After then, rhamnolipids were successfully produced by *Pseudomonas aeruginosa* grown on various waste frying oils, which indicated soybean waste frying oil was the best substrate, produced 9.3 g/L under batch‐fed cultivation with the addition of rhamnolipid precursor (Raza et al., 2006). In this work, we found that *P. aphidis* ZJUDM34 can transform waste cooking oil into MELs, and the yield of MELs is close to that grown on soybean oil substrate at the optimized culture conditions and higher than the yields of sophorolipids and rhamnolipids.

Generally, the fatty acid compositions in MEL structure determine the characterization of MELs in actual application. The total fatty acid profile of purified MELs from WCO showed that fatty acid groups mainly composed of C18:n, followed by C16:0 (Table 4). Such profile was different from that found in MELs grown on soybean oil, preferentially C8:0 and C10:n, as also previously reported (Rau, Nguyen, Schulz et al., 2005). Meanwhile, the CMC values of MEL produced from soybean oil and WCO were both 20.00 mg/L. It implies that the use of WCO substrate represents the same characteristics as soybean oil in considering the surface property. Naturally, the replacement of major fermentation substrate with waste cooking oil remains the same chemical bioactivity.

In summary, this work shows that waste cooking oil can be used as an alternative substrate for MEL production from *P. aphidis* ZJUDM34, which helps to lower the production cost of MELs, facilitating its production and application in the commercial use.

## ETHICAL STATEMENT

This study does not involve any human or animal testing.

## CONFLICT OF INTEREST

The authors declare that they do not have any conflict of interest.
